# Dermatomycosis Caused by Non‐Dermatophyte Agents; Diagnosis Based on Molecular Identification

**DOI:** 10.1002/mbo3.70341

**Published:** 2026-06-21

**Authors:** Fatemeh Zahra Ranjbar Golafshani, Ailin Akhondzadeh, Ali Dastbaz Momtaz, Zeynab Aryanian, Firoozeh Kermani, Erfan Ghaffari Lashkenari, Saeid Mahdavi Omran

**Affiliations:** ^1^ Student Research Committee Babol University of Medical Sciences Babol Iran Babol Iran; ^2^ Department of Parasitology and Mycology Faculty of Medicine, Babol University of Medical Sciences Babol Iran; ^3^ Infectious Diseases and Tropical Medicine Research Center, Health Research Institute, Babol University of Medical Sciences Babol Iran; ^4^ Autoimmune Bullous Diseases Research Center Razi Hospital Tehran University of Medical Sciences (TUMS) Tehran Iran

**Keywords:** dermatomycosis, DNA sequencing, fungal skin infections, molecular identification, non‐dermatophytic fungi

## Abstract

Dermatomycoses are a superficial fungal infections affecting of the skin, hair and nails. While dermatophytes are recognized as the primary causative agents of fungal skin infections, the significance of yeasts and molds in this context warrants greater attention. The present study analyzed samples from patients suspected of non‐dermatophyte superficial infections who were referred to dermatology clinics in Babol. The collected samples underwent direct microscopic examination and culture. Identification of the isolates was performed using standard mycological laboratory techniques, including morphological assessments and slide culture. Molecular identification was achieved through DNA extraction and subsequent sequence analysis of the ITS1‐5.8S rDNA‐ITS4 region. Demographic analysis of 25 patients indicated a predominance of females (72%). The average age of the patients was 52.2 years. Clinical manifestations exhibited considerable variability, with the most prevalent symptoms including pruritus, erythema and scaling. The most frequently affected sites included the feet, nails and groin. Although *Candida albicans* was the predominant isolate identified, a diverse array of other fungal species was also recognized. The variability seen in patients' demographics, clinical signs and fungal causes highlights the complexity of these infections. Correctly identifying fungal species is essential for proper patient treatment.

## Graphical abstract

This study greatly enhances the growing evidence highlighting the role of non‐dermatophyte fungi in causing dermatomycoses. The variation in patient demographics, clinical signs, and fungal causes demonstrates the complexity of these infections. More research is needed to understand how predisposing factors, environmental exposures and the disease process interact in dermatomycoses caused by non‐dermatophyte and less common fungi. These results underscore the importance of increasing clinician awareness of the wide range of possible pathogens and the vital need for precise mycological diagnosis to ensure effective patient treatment.

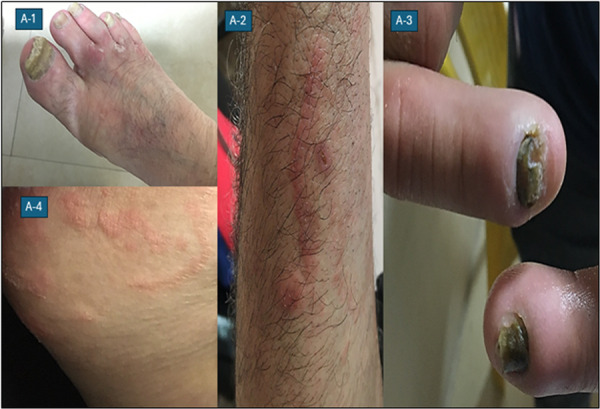

## 1 Introduction

Dermatomycoses are superficial fungal infections that impact the skin, hair and nails. These infections are estimated to affect approximately 20%–25% of the global human and animal populations, with a notably higher prevalence in tropical and subtropical regions (Jaishi et al. 2022). These infections are attributed to a diverse of organisms, including yeasts, dermatophyte species, hyaline and dematiaceous molds (Kruithoff et al. 2024). While dermatophytes are widely recognized as the primary etiological agents of dermatomycoses, the importance of yeasts and molds in this context should not be underestimated (Nenoff et al. 2014). The frequency of these infections and the distribution of their causative agents can vary significantly due to geographical region, population migration patterns, climate, socioeconomic status, lifestyle and cultural factors (Li et al. 2024). Opportunistic mycoses are relatively uncommon and primarily present as cutaneous infections in animals. Some fungi can cause superficial infections when they accidentally penetrate and colonize injured skin, especially in hosts with immune deficiencies (Belmokhtar et al. 2024). Although infections caused by these organisms are less common, they present substantial challenges regarding both diagnosis and treatment. The clinical manifestations of fungal infections due to yeasts and molds often similar to dermatophyte infections, which complicates accurate diagnosis and and potentially leading to inappropriate treatment decisions (Belmokhtar et al. 2024; Khan et al. 2021).

A definitive diagnosis of dermatomycoses caused by yeasts and molds typically requires specialized testing procedures, including direct microscopic examination and fungal culture. However, these assessments may not be readily available to all practitioners due to facility and time constraints (Howell 2023). Furthermore, this fungi are characterized by a significant degree of biodiversity, with each species possessing the ability to respond differently to therapeutic interventions and This complexity challenges in both diagnostic and therapeutic processes (Howell 2023; Tampieri 2004). Accurate reporting is of utmost importance and It is essential that both patients and healthcare practitioners diligently document clinical signs and symptoms. This study thoroughly molecular identification of non‐dermatophytic and rare etiological agents that cause dermatomycoses by using DNA sequencing methods.

## 2 Materials and Methods

### 2.1 Study Design and Sampling

This study was conducted by a group of patients with skin fungal infections in the Diagnostic Center of Skin Diseases in Babol city who sought treatment at selected dermatology clinics over 1 year, and the aim was to identify patients with non‐dermatophytic fungal infections. Therefore, all positive cultures of dermatophytes were excluded from the study. Non‐dermatophytic dermatomycosis was sampled again under sterile conditions to confirm the diagnosis. Participants were included if they exhibited clinical symptoms indicative of acute skin infections, such as pruritus, erythema and scaling. The study received ethical approval IR.MUBABOL.HRI.REC.1403.216 from the Ethical committee of Babol University of Medical Sciences, and informed written consent was obtained from all participants before entering the study. To begin sampling, the scalpel, razor and forceps were disinfected with ethanol alcohol and then strilized by heat. In addition, the patient's skin and nails were disinfected with alcohol before sampling and then samples were taken by scrapping off skin and nail. Infections were classified as non‐dermatophytic based on the initial physician diagnosis, as well as the findings from microscopic examination and culture.

### 2.2 Diagnostic Approach

**Figure 1:**
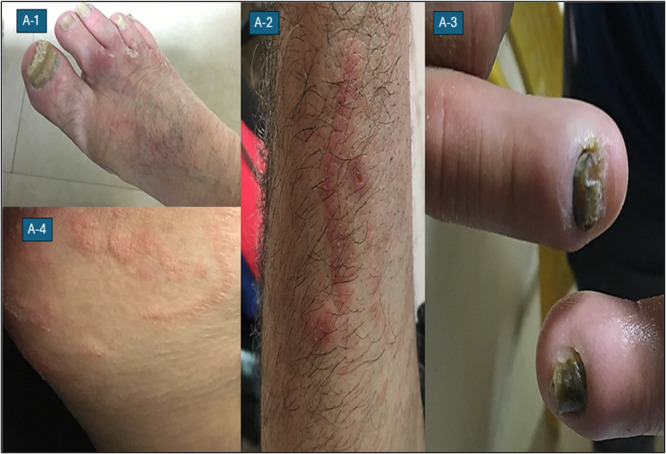
Direct microscopy of skin and nail scraping samples in KOH solution with ×40 magnification. (A) Fungal septate hyphae in skin scraping sample were seen. (B) Many fungal septate and branching hyphae in skin scraping sample were seen. (C) Yeast cells, germ tube and psudohyphae in nail sample were seen.

The obtained samples underwent direct microscopic examination using 10% potassium hydroxide (KOH) for skin scrapings and 30% KOH for nail samples (Figure 1). All samples were taken from patients in two culture mediums of Dextrose Agar (SDA) (Merck, Germany) supplemented with 0.05 g/L chloramphenicol to inhibit bacterial growth and SDA supplemented with 0.05 g/L chloramphenicol and with 0.5 g/L cycloheximide. Inoculation was conducted using spot method across three distinct areas of the agar plate. The inoculated plates were incubated at 28°C for a maximum duration of 4 weeks, with daily observation conducted to monitor the growth of fungal colonies. Following an appropriate incubation period (a minimum of 48 h and a maximum of 4 weeks), the developed colonies were assessed for macroscopic characteristics, which included the coloration of both the Surface and reverse surfaces of the colony, texture (classified as yeast‐like, moldy, or pasty), colony shape, margin, and growth rate.

### 2.3 Identification of Isolates

The identification of the isolates was conducted using standardized mycological laboratory procedures, which included morphological examination on SDA and Potato Dextrose Agar (PDA) (Difco, Detroit, MI, USA). Slide culture or microculture techniques were employed to analyze the morphology of conidia and hyphae on PDA. For microscopic analysis, slides were prepared from each colony exhibiting different morphological characteristics, utilizing the lactophenol aniline blue staining method. Microscopic features, including the presence of yeast and the type and morphology of hyphae (whether septate or non‐septate), as well as the presence or absence of reproductive structures (such as conidiophores, conidia and ect.), were meticulously analyzed and documented.

### 2.4 Molecular Identification

DNA was extracted utilizing the phenol/chloroform method (Yamada et al. 2002). The ITS1‐5.8SrDNA‐ITS4 region was amplified for subsequent sequence analysis. Polymerase chain reaction (PCR) mixture was prepared in a volume of 25 μL and the primers ITS1 (5′‐TCC GTA GGT GAA CCT GCGG‐3′) and ITS4 (5′‐TCC TCC GCT TAT TGA TAT GC‐3′) were used. The PCR cycling conditions were defined and then PCR products were loaded onto a 1.5% agarose gel and stained with 0.5 μg/mL Safestain, subsequently visualized using a gel documentation system (UVITEC, UK) and photographed. The PCR products were purified, and cycle sequencing reactions were conducted in the forward direction (Bioneer, South Korea). The PCR products were sequenced, and the FASTA file sequences were modified, aligned, and submitted to Gene Bank (https://www.ncbi.nlm.nih.gov/genbank/) to obtain accession numbers, after which they were compared to other reference strains from the different sequences. The level of similarity above 99% was selected as the species.

## 3 Results

The analysis of 25 patients' demographics revealed a significant gender disparity, with females constituting 72% and males comprising 28%. The ages of the patients ranged from 26 to 82 years, with a mean age of 52.2 years and a median age of 52 years.

**Figure 2:**
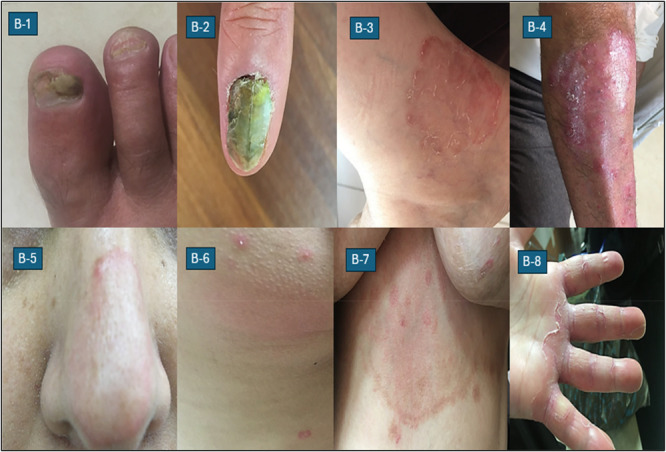
Clinical manifestations of dermatomycosis lesions caused by filamentous fungi. A‐1 *P. chrysogenum* A‐2 *E. nigrum* A‐3 *S. strictum* A‐4 *A. phoenicis*.

**Figure 3:**
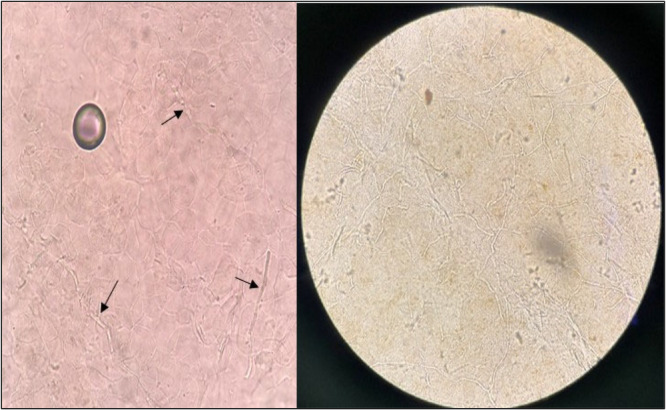
Clinical manifestations of dermatomycosis lesions caused by yeast fungi. B‐1 *R. mucilaginosa* B‐2 *R. macerans* B‐3 *S. roseus* B‐4 *C. catenulata* B‐5 *C. albicans* B‐6 *C. dermatis* B‐7 *C. dermatis* B‐8 *N. glabratus*.

The clinical presentation of dermatomycoses showed considerable variability, and lesions appeared in several anatomical sites. The duration of symptoms ranged from a few weeks to several years, indicating the potential for both acute and chronic course of these infections. Assessment of clinical feautures revealed that pruritus was the most prevalent symptom, observed in 84% of patients (21 out of 25), followed by erythema in 64% (16 out of 25) and scaling in 60% (15 out of 25). The anatomical locations of lesions varied, with the feet, nails and groin being the most commonly involved sites, each observed in 4 cases. Other site included the axillae (2 cases), forearms (2 cases), and several other sites with single occurrences, indicating diversity in the areas where dermatomycosis manifested (Figures 2 and 3).

The duration of symptoms before clinical manifestation showed substantial variability. The mean duration of symptom onset was 16.22 months, while the median duration was 6 months. Several potential risk factors for dermatomycoses were identified in the patient data. A positive history of similar symptoms in relatives was reported in some cases, suggesting the possibility of familial transmission or shared environmental exposures. Exposure to public facilities such as gyms and pools was also noted for some patients, as these environments are known to facilitate the transmission of certain fungal pathogens. The onset durations ranged from 15 days to 15 years, while most patients experienced symptoms for a shorter duration, a subset experienced symptoms for a considerably longer period.

Mycological analysis revealed *Candida albicans* as the predominant isolate recovered from four cases. However, the etiological considerable diversity, with a range of other fungal species identified. These included *Epicoccum nigrum*, *Candida parapsilosis*, *Sarocladium strictum*, *Aspergillus phoenicis*, *Cutaneotrichosporon dermatis*, *Nakaseomyces glabratus*, *Rhodotorula mucilaginosa*, *Sporobolomyces roseus*, *Candida catenulata (Diutina catenulata)*, *Penicillium chrysogenum*, *Bullera alba*, *Rhodotorula macerans* (*Cystofilobasidium macerans)* and *Trichosporon asahii* (Table 1).

**Table 1:** Patient demographics, clinical characteristics, and mycological findings.

Sex	Age	PMH	Residency	Location of lesion	Itching	Erythema	Scaling	Onset of symp. (month)	Symp. in relatives	Gym	Pool	Genus and species	Accession number
F	52	Blood clot in the leg vein	C	Foot nail	N	N	P	3	N	N	N	*C. albicans*	PV070679
M	82	DM, IHD, HLP, Exposed to fluvial water Poultry keeping	R	Foot nails	N	N	P	4	N	N	N	*P. chrysogenum*	PV082177
F	26	—	C	Axilla	P	P	P	5	N	N	P	*N. glabratus*	PV072424
F	59	DM2 Insulin‐dependent (15 y.), HTN	R	One hand	N	P	P	48	N	N	N	*N. glabratus*	PV074709
F	38	—	R	Knee, hand	P	P	P	12	P	N	N	*R. mucilaginosa*	PV074284
F	63	—	C	Nose	P	P	N	3	N	N	N	*C. albicans*	PV072422
F	63	—	C	Groin	P	P	N	3	N	N	N	*C. albicans*	PV072426
M	57	Deaf‐mute	R	Forearm	P	P	P	18	P	N	N	*C. catenulata*	PV073812
F	41	—	C	Buttock, Groin, Knee	P	N	N	1	P	P	N	*C. dermatis*	PV072847
M	39	—	C	Hand	P	P	P	P	P	P	P	*E. nigrum*	PV075238
F	39	Hypothyroidism	C	Genital	P	P	N	6	P	N	N	*C. albicans*	PV072431
F	45	Post‐Pregnancy Depression	R	Under chest, Groin, buttocks, Axilla	P	P	P	6	N	N	N	*C. parapsilosis*	PV074065
F	45	Mental retard	R	Under the chest, axilla, groin	P	P	P	5	P	N	N	*C. dermatis*	PV072561
M	57	Albuminuria	C	Forearm, buttock, Nail	P	P	N	2 weeks	P	P	N	*S. strictum*	PV074343
M	36	Kidney stone	C	Groin	P	P	P	3	N	N	N	*S. roseus*	PV074876
F	67	RA	R	Axilla, Foot	P	P	P	2	N	N	N	*S. roseus*	PV073581
F	66	Epilepsy, asthma, HTN, bilateral knee surgery	R	Under the chest, axilla, groin	P	P	N	5	P	N	N	*T. asahii*	PV075091
F	39	—	C	Genital	P	P	P	7	P	P	P	*C. parapsilosis*	PV075071
M	72	CABG	R	Foot nail, Pedis	P	N	N	3	N	N	N	*B. alba*	PV073981
F	40	—	C	Under the chest, axilla,	P	N	N	5 years	N	N	N	*A. phoenicis*	PV070680
F	56	DM	C	Groin, buttock				6	N	N	N	*C. catenulata*	PV075033
M	72	CABG	R	Foot nail, Pedis	N	N	N	3	N	N	N	*R. mucilaginosa*	PV072587
F	44	—	C	Foot nail	N	N	N	18	N	N	P	*C. albicans*	PV075677
F	48	HTN	C	Foot nail	N	N	N	3	N	P	N	*R. mucilaginosa*	PV075240
F	64	HLP	C	Nail	N	N	N	15 years	N	N	P	*R. macerans*	PV075236

Abbreviations: CABG, coronary artery bypass grafting; C, city; DM, diabetes mellitus; DM2, Type 2 diabetes mellitus; F, female; HLP, hyperlipidemia; HTN, hypertension; IHD, ischemic heart disease; M, male; N, negative; P, positive; R, rural; RA, rheumatoid arthritis; Symp., symptoms.

## 4 Discussion

This study provides insights into the etiology of dermatomycoses in the studied population, highlighting several key findings that warrant further discussion.

The observed higher prevalence of dermatomycoses in women (72%) compared to men (28%) represents a significant finding, as reported by Araya et al. (2021) (Araya et al. 2021). Potential explanations for this disparity may encompass hormonal influences, chenges in skin physiology, hygiene practices and occupational or lifestyle exposures that could contribute. For example, specific cosmetic or skincare products may establish a microenvironment on the skin that promotes fungal overgrowth in women (Petranyuk et al. 2022).

The age distribution of patients ranged from 26 to 82 years, with a mean and median age of approximately 52 years, indicating that dermatomycoses caused by non‐dermatophyte fungi are not confined to any specific age group. This data suggests that dermatomycoses within this cohort impact adults across a wide age spectrum (Chanyachailert et al. 2023). The broad age range observed in this study likely reflects the diverse risk factors and underlying conditions that may predispose individuals to opportunistic fungal infections. For example, older adults may experience age‐related alterations in immune function and skin health, whereas younger individuals may be exposed to fungi through specific activities or environmental contexts (Jaishi et al. 2022).

The clinical manifestation of dermatomycoses in this study showed considerable variability. While pruritus emerged as the most common symptom, both the presence and severity of erythema and scaling demonstrated significant variation among patients. Lesions were identified across a range of anatomical sites, with the feet, nails and groin being particularly prevalent. This clinical heterogeneity poses a considerable diagnostic challenge, as infections caused by non‐dermatophyte fungi often mimic other dermatoses, including dermatophyte infections (Chanyachailert et al. 2023). This highlights the critical significance of mycological investigations, including microscopy and culture techniques, in ensuring accurate diagnosis and effective management (Jaishi et al. 2022).

The mycological findings of this study revealed a diverse array of yeasts and molds was also identified. The prevalence of *Candida* and *Rhodotorula* spp. aligns with their recognized status as prevalent opportunistic skin pathogens (Jaworek et al. 2025). Factors including antibiotic administration, immunosuppression, and alterations in the skin microbiome may elevate the risk of cutaneous candidiasis (Silva et al. 2014; Jerez Puebla 2012). The isolation of multiple *Candida* species highlights the need for accurate species identification, as antifungal susceptibility can differ among them (Araya et al. 2021). The detection of various molds, including *Epicoccum*, *Aspergillus*, and *Scopulariopsis*, calls for further investigation (Bitew et al. 2022; Nenoff et al. 2022). These fungi are primarily environmental saprophytes and are less frequently linked to skin infections than dermatophytes or *Candida*. Their presence suggests that they may act as opportunistic pathogens, particularly in individuals with weakened immune systems (Bitew et al. 2022; Hube et al. 2015).

The diversity of fungal isolates holds significant therapeutic implications. Specifically, infections caused by yeasts and molds often exhibit clinical features that closely resemble those of dermatophyte infections. This resemblance can result in misdiagnosis and, consequently, inappropriate therapeutic interventions (Jaworek et al. 2025). The clinical presentation of dermatomycosis lesions caused by filamentous fungi and yeasts exhibits minimal variation. Furthermore, this lack of discernible difference persists even among different genera of yeast fungi, thereby complicating the diagnostic process (Jaishi et al. 2022). One of the principal challenges in the diagnosis and management of fungal infections lies in the similarities of clinical manifestations exhibited by various fungal species. Non‐dermatophyte fungi frequently present distinct antifungal susceptibility profiles in comparison to dermatophytes. Consequently, precise identification of the causative agent is essential for the selection of the most effective antifungal therapy and for minimizing the risk of treatment failure (Nenoff et al. 2022; López‐Jodra and Torres‐Rodríguez 1999).

Relying exclusively on the visual assessment of skin lesions or the outcomes of traditional fungal cultures may hinder the accurate identification of the specific fungal species responsible for the infection (Fang et al. 2023). For example, many yeasts exhibit morphologically similar colonies in culture that are impossible to distinguish based on primary macroscopic or even basic microscopic features. Similarly, certain molds display analogous morphological features. This limitation in the accurate identification of fungal species can have substantial implications for treatment (Otašević et al. 2018). Distinct fungal species demonstrate differential susceptibilities to antifungal agents; therefore, a medication that is efficacious against one species may be ineffective against another. Inaccurate identification of the fungal species, coupled with the subsequent selection of inappropriate antifungal agents, can lead to suboptimal treatment responses, recurrent infections, and chronic conditions (Pagano and Fernández 2025). This not only exacerbates patient suffering but also increases treatment costs. In contrast, molecular diagnostic techniques, particularly DNA sequencing, enable the precise identification of fungal species. By analyzing the genetic sequence of the fungus, these methodologies can accurately ascertain its species, even when its morphological or culture characteristics may be misleading (Posadas‐Cantera et al. 2024). The utilization of molecular diagnostic methods allows clinicians to consider the epidemiological pattern of specific fungal species involved in the infection. This strategy increases the probability of successful treatment outcomes and reduces the risk of recurrent infections (Otašević et al. 2018; Naik et al. 2024). Consequently, molecular diagnostics are essential for the effective management of dermatomycoses, especially in instances where infections fail to respond to conventional therapies or demonstrate recurrence (Naik et al. 2024).

Given the enhanced sensitivity and specificity of molecular methods for fungal detection (Otašević et al. 2018) it can be said that the most reliable results are obtained from a comination of direct microscopy examination, culture and PCR (Nenoff et al. 2022).

### 4.1 Limitations

The constrained sample size restricts the generalizability of the findings. Conducting a larger, multicenter investigation would improve our comprehension of the epidemiology and etiology of non‐dermatophyte dermatomycoses.

## 5 Conclusion

Non‐dermatophyte cutaneous mycoses present clinical manifestation similar to dermatophyte infection. Even direct microscopy examination and culture require high precision to avoid being considered as colonization or contamination. In addition, the integration of direct microscopy examination, culture and molecular methods for accurate identification is necessary. These results underscore the importance of increasing clinician awareness of the wide range of possible pathogens and the vital need for precise mycological diagnosis to ensure effective patient treatment.

## Author Contributions

**Fatemeh Zahra Ranjbar Golafshani:** writing – original draft, conceptualization, data curation, methodology. **Ailin Akhondzadeh:** data curation, resources, conceptualization, visualization, validation, writing – review and editing. **Ali Dastbaz Momtaz:** investigation, writing – review and editing, methodology, software. **Zeynab Aryanian:** supervision, writing – original draft, data curation, resources. **Firoozeh Kermani:** writing – original draft, writing – review and editing, validation, visualization. **Erfan Ghaffari Lashkenari:** validation, data curation, resources, investigation,writing – review and editing. **Saeid Mahdavi Omran:** supervision, project administration, writing – original draft, conceptualization, formal analysis, funding acquisition. All authors have read and approved the final manuscript.

This article is the outcome of a research project approved under code 724135878, which received funding from Babol University of Medical Sciences.

## Ethics Statement

Written informed consent was obtained from the patients before the commencement of this research. Babol University of Medical Sciences conducted the study in compliance with the ethical guidelines specified under code IR.MUBABOL.HRI.REC.1403.216.

## Consent

The authors affirm that human research participants have given informed consent for publication.

## Conflicts of Interest

The authors declare no conflicts of interest.

## Data Availability

The data that support the findings of this study are available on request from the corresponding author. The data are not publicly available due to privacy or ethical restrictions.
